# Adaptation by the Brown Planthopper to Resistant Rice: A Test of Female-Derived Virulence and the Role of Yeast-like Symbionts

**DOI:** 10.3390/insects12100908

**Published:** 2021-10-06

**Authors:** Finbarr G. Horgan, Ainara Peñalver Cruz, Arriza Arida, Jedeliza B. Ferrater, Carmencita C. Bernal

**Affiliations:** 1 EcoLaVerna Integral Restoration Ecology, Bridestown, Kildinan, T56 P499 Country Cork, Ireland; 2 Escuela de Agronomía, Facultad de Ciencias Agrarias y Forestales, Universidad Católica del Maule, Casilla 7-D, Talca 3460000, Maule, Chile; 3 International Rice Research Institute, Makati 1226, Metro Manila, Philippines; ainara.penalver@agrocampus-ouest.fr (A.P.C.); a.arida@gmail.com (A.A.); jedeliza.ferrater@ucr.edu (J.B.F.); c.bernal@irri.org (C.C.B.); 4 Institut de Génétique, Environnement et Protection des Plantes (UMR), Agrocampus-Ouest, 2 Rue André le Nôtre, 49045 Angers, France; 5 Department of Entomology, University of California Riverside, 3401 Watkins Drive, Riverside, CA 92507, USA

**Keywords:** *BPH3*, *BPH32*, Delphacidae, endosymbionts, Heteroptera, honeydew, host plant resistance, Integrated Pest Management, resistance management, rice breeding

## Abstract

**Simple Summary:**

The brown planthopper (*Niliparvata lugens* [Stål]) feeds on rice in Asia. Rice breeders have developed varieties with resistance to the planthopper; however, planthoppers quickly adapt to these varieties (called ‘virulence adaptation’). We examined the potential role during adaptation of endosymbionts that are passed by the female through the egg. We adapted planthoppers by exposure to resistant rice (IR62) for >20 generations, and crossed males or females from these adapted populations with non-adapted males and females that were reared on susceptible rice. Virulent males and females contributed to the virulence of their offspring as revealed through faster development rates, higher growth rates, greater numbers of female offspring, and an increased longevity of progeny on IR62 compared to the progeny of avirulent planthoppers on IR62; however, the female contribution to virulence was greater than that of the male. Reducing densities of YLS in adult planthoppers through heat treatment reduced their capacity to gain weight on IR62, irrespective of the virulence of parents, thereby suggesting that YLS derived from the female parent play a role in the vertical transfer of virulence; however, the effects were weak and IR62 seedlings were still more affected by heat-treated planthoppers with a virulent female parent than by heat-treated planthoppers with a virulent male parent or with avirulent parents only. Virulence is a complex state with multiple underlying mechanisms, some of which are derived only from the female parent.

**Abstract:**

The adaptation by planthoppers to feed and develop on resistant rice is a challenge for pest management in Asia. We conducted a series of manipulative experiments with the brown planthopper (*Nilaparvata lugens* (Stål)) on the resistant rice variety IR62 (*BPH3*/*BPH32* genes) to assess behavioral and bionomic changes in planthoppers exhibiting virulence adaptation. We also examined the potential role of yeast-like symbionts (YLS) in virulence adaptation by assessing progeny fitness (survival × reproduction) following controlled matings between virulent males or females and avirulent males or females, and by manipulating YLS densities in progeny through heat treatment. We found virulence-adapted planthoppers developed faster, grew larger, had adults that survived for longer, had female-biased progeny, and produced more eggs than non-selected planthoppers on the resistant variety. However, feeding capacity—as revealed through honeydew composition—remained inefficient on IR62, even after 20+ generations of exposure to the resistant host. Virulence was derived from both the male and female parents; however, females contributed more than males to progeny virulence. We found that YLS are essential for normal planthopper development and densities are highest in virulent nymphs feeding on the resistant host; however, we found only weak evidence that YLS densities contributed more to virulence. Virulence against IR62 in the brown planthopper, therefore, involves a complex of traits that encompass a series of behavioral, physiological, and genetic mechanisms, some of which are determined only by the female parent.

## 1. Introduction

The brown planthopper, *Nilaparvata lugens* (Stål), is one of the principal pests of rice throughout Asia [1]. Research to reduce planthopper-related yield losses in rice has focused on restoring ecosystem resilience by enhancing natural biological control and reducing the vulnerability of rice plants [2,3,4]. This includes the breeding of modern, high-yielding varieties with resistance to the pest [5,6]. Several traditional rice varieties and wild rice species have natural resistance to planthopper populations and over 40 planthopper-resistant gene loci are currently known [6]. A range of rice varieties with resistance to the brown planthopper have now been developed using traditional and wild rice donors and some have been released to farmers’ fields [4]. However, the utility of resistant varieties and their underlying resistance genes is limited by the ability of planthoppers to adapt to and overcome host defenses [4,7,8,9,10]. Evidence suggests that planthopper populations include rare individuals that are virulent to one or more resistance genes. These individuals, called ‘forerunners’, can rapidly build up populations in the absence of competition with avirulent conspecifics in fields of resistant rice. Virulence adaptation can occur in as little as 15 generations (2 years at tropical latitudes) [4]. 

The mechanisms underlying virulence in planthoppers against resistant rice have received only limited research attention. Virulent planthoppers generally demonstrate similar feeding capacity, development rates, weight gain, and reproductive output on rice varieties with and without major resistance genes [8,9,10,11]. This virulence can be partly due to changes in planthopper behavior on the resistant host, including changes in preferred feeding sites [12], or due to an increased consumption of fluids that are transported in the xylem [13,14]. There is some evidence that virulence may be associated with virulence genes. Kobayashi et al. (2014) [15] present evidence from breeding experiments with virulent and avirulent planthoppers for a single recessive gene controlling the planthopper’s capacity to feed on rice with the *BPH1* resistance gene. In a similar study, Jing et al. (2014) [16] found that virulence against the same *BPH1* gene was associated with a few major resistance genes governing different aspects of planthopper fitness. Differences in the genetic mechanisms detected by these authors, despite using the same study system (planthoppers on *BPH1* resistant rice), suggest that there are several genetic and mechanistic pathways to virulence. Some authors have also suggested that virulence may be derived from endosymbiotic bacteria or yeasts associated with the planthopper’s digestive system [7,17,18,19]. These endosymbionts would adapt more quickly than their mutualist planthopper hosts to overcome toxins produced by resistant rice, or they may enhance planthopper survival and development on resistant varieties by providing essential vitamins and amino acids to the mutualist that are otherwise unavailable from the resistant plant. Much of the research into the role of endosymbionts in planthopper adaptation has focused on the yeast-like symbionts (YLS).

Yeast-like symbionts (Class: Pyrenomycetes; Subphylum: Ascomycotina) include two or more species [20]; YLS occur at every developmental stage of the brown planthopper, with the highest numbers in brachypterous females at about the time of egg laying [17,20,21,22]. These symbionts conserve nitrogenous wastes from planthopper digestion to produce available nutrients for the planthopper [23,24]. The reduction in YLS numbers through heat treatment to produce aposymbiotic planthoppers has been associated with poor development of planthopper embryos, slow development of nymphs, and failure to develop beyond the fifth instar [25,26,27]. Research has tracked changes in the abundance of YLS during planthopper adaptation. Lu et al. (2004) [18] found YLS numbers to decrease when planthoppers were switched from a susceptible to a resistant rice variety; numbers then gradually built up over successive generations on the resistant host. Furthermore, by comparing enzyme activity in aposymbiotic and symbiotic planthoppers on different rice hosts, these authors suggested that YLS made different contributions to amino acid utilization in planthoppers that were continuously reared on the different rice hosts. In a study by Chen et al. (2011) [7], YLS were associated with improved nymph performance on resistant rice, but the authors suggested that the continuous exposure of the planthoppers to a single host variety (i.e., benign environment) possibly led to YLS becoming a drain on the mutualist host—suggesting that YLS play a role in the adaptability of planthoppers to complex and changing environments. 

More recent research has verified that YLS densities do change when planthoppers are switched between varieties (including from one susceptible to another susceptible variety), but that the direction of change is not consistent (i.e., densities might increase or decrease depending on the exposed variety) [14,28]. Furthermore, YLS were not a drain on the mutualist host even after 20+ generations of continuous rearing on some varieties [28]. Apparent YLS modifications (i.e., density changes, roles in planthopper nutrition) during feeding by the mutualist host on resistant rice, as revealed through selection experiments [7,14,18], could be direct responses to rice defense mechanisms that enhance planthopper survival, but they could also be the indirect effects of depleted nutrient acquisition by the host (i.e., planthopper starvation). Therefore, research on the role of endosymbionts during virulence adaptation remains largely inconclusive. Because YLS are transmitted to progeny through the egg [22,29,30,31], any benefits to virulent planthoppers that come from changes to YLS can only be derived from the female parent. Therefore, the role of YLS in virulence adaptation could be substantiated through controlled mating experiments. 

**Figure 1:**
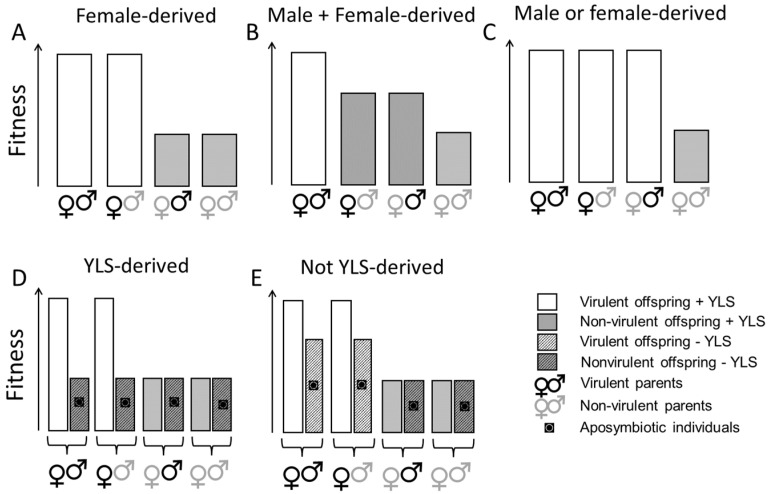
Study predictions based on the vertical transmission of endosymbionts through the egg and a possible role for YLS in virulence adaptation. If endosymbionts determine virulence, then the progeny of virulent females will also be virulent, irrespective of the male parent (**A**). If the virulent male parent also determines virulence, then progeny virulence will respond in an additive manner to having one or two virulent parents (**B**). If virulence is determined by either parent (i.e., in the case of a major resistance gene), then progeny with one or two virulent parents, of either sex, will be virulent (**C**). Where virulence is female-derived, the role of YLS and other vertically transmitted endosymbionts can be assessed by comparing aposymbiotic and symbiotic planthoppers. Where the relative fitness of progeny with virulent or avirulent female parents is affected by removing endosymbionts (aposymbiotic), then the symbionts play a role in virulence adaptation (**D**). Where relative fitness patterns are maintained despite planthoppers being aposymbiotic, then the endosymbionts play little role in virulence adaptation (**E**).

In this study, we conducted a range of controlled mating experiments to examine the roles of the male and female parents in virulence adaptation. We used planthoppers from colonies with virulence to the highly resistant rice variety IR62 [14,28,32] and from avirulent colonies that were continuously reared on a susceptible variety. We predicted that if YLS play a role in virulence adaptation, then planthoppers with virulent female parents will demonstrate greater virulence than planthoppers with avirulent female parents or with a virulent male parent only. Furthermore, where YLS are depleted by heat treatments, then planthoppers with a virulent female parent will exhibit similar avirulence when compared to planthoppers with avirulent female parents or planthoppers with a virulent male parent only. By comparing the fitness of progeny on a resistant host, mating experiments, together with the manipulation of YLS densities, could therefore elucidate the relative roles of genetics and endosymbionts in determining virulence as outlined in Figure 1. To our knowledge, this is the first study of YLS in the brown planthopper to manipulate virulent and avirulent mating pairs and evaluate the effects on planthopper progeny.

## 2. Materials and Methods

### 2.1. Plant Materials

We used TN1 as a food plant for avirulent colonies; this rice variety is highly susceptible to the brown planthopper [14,18,28]. The variety IR22, a further susceptible variety that is closely related to IR62 [14,18,32], was used in some experiments as an intermediate host where planthoppers or their progeny were moved between varieties. We used IR62 as the resistant host. IR62 was released by the International Rice Research Institute (IRRI) in 1984 [32]. The variety is resistant to brown planthopper populations throughout South and Southeast Asia [33]. Furthermore, the variety has maintained its resistance despite planting for over 30 years in some parts of Cambodia and Mindanao [34]. This broad spectrum (affecting several planthopper populations) and durable resistance of IR62 is likely due to ≥2 genes derived from the Indian landrace PTB33 during breeding [32,33]. As such, IR62 is thought to contain the *BPH3* gene and/or the *BPH32* gene [32,33,34]. Seed of all three varieties were acquired from the IRRI-germplasm bank. All plants were initially seeded in saturated paddy soil in the greenhouse and transplanted to pots at 7–10 days after sowing (DAS). Plants were watered daily and received no fertilizer or pesticide treatments. 

### 2.2. Planthopper Colonies

Planthoppers were obtained from colonies reared at IRRI, Philippines. Planthoppers were initially collected in 2004 from rice fields in Laguna Province. The founder population (ca 500 individuals) was placed in wire mesh cages of 120 × 60 × 60cm (H × W × L) under greenhouse conditions (temperatures 25–37 °C; 70–90% relative humidity (RH), 12 h–12 h day–night (D–N)) and was continuously reared on ≥30 DAS TN1 plants. After 32 generations, the IRRI colony was introgressed with newly wild caught individuals (ca 500) from rice fields in Santa Cruz, Laguna. After a further 5 generations, adult planthoppers were taken from the main cage and placed on IR62 (≥30 DAS) in two separate cages—designated colony A (IR62A) and colony B (IR62B). At the same time, a new colony of planthoppers reared on TN1 was initiated using 500 adults. The original colony with TN1 as a natal host was designated colony A (TN1A) and the second, colony B (TN1B). These were maintained for several generations without any further introgression of wild caught individuals. Feeding plants for all colonies were replaced every two weeks. We maintained two planthopper colonies on each host plant to reduce inbreeding potential during our experiments; however, we estimate that each colony produced a minimum of 5000 nymphs every 30 days, with colonies reaching ca 12,000 nymphs per cage after 20 generations. At the time that experiments were initiated, the colonies had been continuously reared on their natal hosts for at least 20 generations. 

### 2.3. Adaptation to IR62 after 20+ Generations of Continuous Rearing

We conducted a series of bioassays to evaluate the performance of planthoppers from each of the four colonies (IR62-reared A and B, TN1-reared A and B) on IR62 and TN1 rice plants and, thereby, assess the effects of virulence adaptation. 

We recorded nymph survival, the proportion of nymphs that emerged as adults, the proportion of adults that were female, and the dry weight of virulent and avirulent planthoppers on each host using the nymph survival bioassay (see below). The longevity of virulent and avirulent male and female adults was examined on each host using the adult survival bioassay (see below). Egg laying by virulent and avirulent planthoppers was examined on each host using the oviposition bioassay (see below). All bioassays were conducted with potted rice plants using a completely randomized design on a greenhouse bench at ambient temperatures (25–32 °C, 70–90% RH, 12 h–12 h DN). There were 12 replicates (10 for the oviposition bioassay) for each bioassay. The bioassays were conducted as follows:

Nymph survival bioassay: Rice plants were individually sown in # 0 pots (5 × 10 cm, H × diam). After 14 days, the pots were each covered with a cylindrical acetate cage (45 × 10 cm, H × diam) with a mesh top. Recently emerged nymphs (<2 h) were collected using a suction pooter from plants that had been exposed to gravid females but from which all free-living planthoppers had since been removed. Nymphs (10 per plant) were placed in the cages through a slit in the acetate when plants were 21–25 DAS. Nymphs were allowed to feed and develop for 15 days after which the number of survivors were recorded. The planthoppers were collected from the cages using a vacuum sampler and the developmental stages recorded. We used the proportions of nymphs that were adults to represent relative development rates as ‘nymph development to adult’. Adults were also sexed (based on external morphology). The nymphs were then dried in a forced draught oven at 60 °C for 1 week and weighed.

Adult survival bioassay: Rice plants were individually sown in # 0 pots (5 × 10 cm, H × diam) and each covered with a cylindrical acetate cage (45 × 10 cm, H × diam). Fifth instar nymphs were collected from the colonies and placed individually in test tubes, each with a 7 DAS rice seedling, until the nymphs became adults. After emergence, the adults were sexed. The unmated adults were then placed inside the acetate cages (6 females or 6 males per cage) when the plants were 21–25 DAS. Only brachypterous females were used to avoid possible differences in longevity between different winged forms; but males were macropterous. The planthoppers were allowed to feed freely and were monitored, noting the times at which 50% and 100% of the adults had died in the cages.

Oviposition bioassay: Rice plants were individually sown in # 0 pots and individually covered with a cylindrical acetate cage (45 × 10 cm, H × diam). Fifth instar nymphs were collected from colonies and placed individually in test tubes, each with a 7 DAS IR22 rice seedling until the nymphs became adults. After emergence, the adults were sexed. The unmated adults were then placed inside the acetate cages (1 ♀ + 1 ♂ per cage) when the exposed host plants (1R62 or TN1) were 21–25 DAS. To avoid differences in egg loads between different winged forms, only brachypterous females were used; males were predominantly macropterous, with some brachypterous forms. The planthoppers were allowed to mate and lay eggs for 7 days after which the rice plants were destructively sampled. The rice plants (with roots) were individually wrapped in moistened tissue paper inside plastic pouches and placed in a refrigerator (4 °C). The plants were dissected under a stereo microscope (10× magnification) to count the eggs. Egg counting was completed within 5 days of sampling.

We further examined the feeding efficiency of virulent and avirulent planthoppers using the honeydew bioassay (see below). This bioassay was conducted on a laboratory bench (24–28 °C, 70% RH, 12 h–12 h D:–N) using a completely randomized design with 12 replicates. The honeydew bioassay was conducted as follows:

Honeydew bioassay: Pre-oviposition gravid, brachypterous females (12 from each mating combination) that had been starved for 2 h, were each confined to specially-prepared plastic chambers (5 × 10 cm: H × diam—one female per chamber), each located at the base of a 14 DAS potted IR62 plant. Each chamber had a hole at the base and at the top, through which the plant passed. A cotton plug prevented the planthoppers from escaping through the top hole. A Whatman No. 1 filter paper, treated with bromocresol green, was placed at the base of each chamber. Bromocresol green indicates the nature of honeydew as blue-rimmed (alkaline) spots and white-rimmed (acidic) spots representing phloem- and xylem-derived honeydew, respectively [35]. The planthoppers were allowed to feed for 24 h after which the filter papers were collected and the area of each spot measured using Image-J version 1.48 (National Institutes of Health, USA). 

Finally, we estimated YLS densities in virulent and avirulent planthoppers when feeding on IR62 and TN1. Newly hatched planthopper nymphs (1st instars) were collected from the colonies and allowed to feed and develop on 20 DAS rice seedlings (2 per seedling). The seedlings had been individually sown in # 0 pots and were each covered with a cylindrical acetate cage (45 × 10 cm: H × diam). After 10 days, the planthoppers were collected from the plants using a pooter and were weighed (wet weight). The planthoppers were then homogenized in 500 μL physiological saline solution (0.9% NaCl). A single aliquot of 10 μL was then transferred to a hemocytometer cell counter and the YLS (visible ovoid bodies) were counted under a compound microscope (40× magnification) [7]. YLS abundance was divided by the weight of the planthoppers to estimate density.

### 2.4. Effects of Adapted Male and Female Parents on Progeny Fitness

We examined the role of virulent male and female planthoppers during the vertical transmission of virulence to progeny. To do this, we examined the performance of planthopper progeny from each of four mating combinations and exposed to IR62 rice plants. Mating experiments were conducted as follows:

Forty 5th instar planthoppers were collected from each of the four colonies using a pooter. Each planthopper nymph was placed individually into test tubes with a 7 DAS rice seedling (same type as the planthopper natal host) until they became adults. The sex of the newly emerged adults was verified (based on external morphology). Adults were paired and each pair released into cylindrical acetate cages (122 × 22 cm, H × diam) and placed on a greenhouse bench. Each cage had a #6 pot (20 × 24 cm, H × diam.) sown with 20 seed from IR22 and plants were 20 DAS when adults were introduced. The mating pairs were as follows: IR62 ♀ + IR62 ♂; IR62 ♀ + TN1 ♂; TN1 ♀ + IR62 ♂; TN1 ♀ + TN1 ♂. Where pairs included males and females reared on the same rice host variety, then one individual was from colony A and the second from colony B to reduce potential inbreeding. Furthermore, pairings were balanced such that roughly half of the planthoppers representing each host plant and sex combination were from colony A and the other half from colony B. The mated pairs were allowed to oviposit and nymphs were maintained on the IR22 host plants until required for bioassays. The nymphs were collected from the acetate cages using a pooter. Progeny from the controlled mating were used in the honeydew bioassay (see above) conducted on a laboratory bench (24–28 °C, 70% RH, 12 h–12 h D–N), and in the nymph survival bioassay (see above) and adult survival bioassay (see above) conducted on a greenhouse bench (25–30 °C, 70–90% RH, 12 h:–12 h D–N). All bioassays were conducted using a completely randomized design and were replicated 12 times.

We also assessed the reproductive potential of progeny from the four mating combinations. However, to ensure that mating was confined to groups with similar parents, we conducted further controlled mating as follows: About 100× 5th instars were collected as progeny (F1) from each original mating combination and placed individually in test tubes with a 7 DAS IR22 seedling until they emerged as adults. The sex of the adults was then determined. The adults were paired with adults (F1) of the opposite sex taken from another cage. For mating, the F1 females were always descended from females taken from the same original colony (i.e., first mating = IR62A ♀ + IR62B ♂; second mating = F1 ♀ (IR62A ♀ × IR62B ♂) + F1 ♂ (IR62B ♀ × IR62A ♂)). This produced 48 mated pairs from four mating combinations (12 × IR62 F1 ♀ + IR62 F1 ♂; 12 × IR62 F1 ♀ + TN1 F1 ♂; 12 × TN1 F1 ♀ + IR62 F1 ♂; 12 × TN1 F1 ♀ + TN1 F1 ♂). Half of the pairs from each combination had females derived from A colonies and the other half had females derived from B colonies. We assessed reproductive potential using the oviposition bioassay (see above) and the population build-up bioassay (described below) conducted in the greenhouse. For the oviposition bioassay, we also assessed egg viability by examining the embryo condition. The bioassays were conducted using a completely randomized design and were replicated 12 times. The population build-up bioassay was conducted as follows:

Population build-up bioassay: Rice plants (3 per pot) were sown in # 6 pots (20 × 24 cm, H × diam). After 14 days, the pots were each covered with a cylindrical acetate cage (122 × 22 cm, H × diam) with a mesh top. Fifth instar nymphs were collected from colonies and placed individually in tubes with 7 DAS IR22 rice seedlings until the nymphs became adult. After emergence, the adults were sexed. The unmated adults were then placed inside the acetate cages (1 ♀ + 1 ♂ per cage) when the plants were 21–25 DAS. The cages were tended for 20 days. After 20 days, each cage was opened by removing the mesh top, and the planthoppers were collected using a vacuum sampler. The planthoppers were counted and their development stages recorded.

### 2.5. YLS Densities and Planthopper Virulence

Unmated adult (F1), brachypterous, female planthoppers from each mating combination were randomly taken from parent colonies (initially collected as 5th instars) and confined to potted (5 × 10 cm, H × diam) 20 DAS IR62 seedlings in cylindrical acetate cages (45 × 10 cm, H × diam). There were 20 females and cages for each mating combination. Ten cages for each combination were moved to a plant growth chamber at 35 °C for 3 days to generate ‘heat-treated’ or aposymbiotic planthoppers [18]. The remaining cages were randomized on a laboratory bench. After 3 days the plants with heat-treated planthoppers were moved back to the laboratory bench and randomly interspersed with the non-heat-treated planthoppers (henceforth symbiotic). The experiment (4 mating combinations × 2 symbiont treatments × 10 replicates) was set up as a completely randomized design. After a further 5 days, the cages were destructively sampled by placing each female in a separate Eppendorf tube. The rice plants were gently pulled from the soil taking care not to damage the main roots and were placed in paper bags (one plant per bag). The seedlings were dried in an oven at 60 °C for 1 week and weighed. Each planthopper was weighed (wet weight) and YLS densities were estimated as described above.

### 2.6. Data Analyses

We used 2-factor general linear models (GLM) to examine the effects of selection (i.e., natal host) and exposed host on nymph survival, nymph development, nymph weight, honeydew production (total honeydew, xylem-derived, phloem-derived and phloem-derived as a proportion of total honeydew), and YLS density. We used a three-factor GLM to analyze adult longevity with colony, host plant, and sex as main factors. Initially we included colony origin (A or B) as a nested factor in the analyses using categorical variables A, B, C, and D (A and B = IR62, C and D = TN1); however, the nested factor had no statistically significant effect and was consequently removed from the analyses. 

We used GLM to analyze honeydew production (total honeydew, phloem-derived as a proportion of total) by FI females derived from controlled mating, and survival, nymph development, female prevalence, and nymph biomass from the nymph survival bioassay. Adult longevity was examined using a repeated measures GLM with the time to 50% and 100% mortality of individuals in each cage as the repeated measure and mating combination and adult sex as main factors. From the *oviposition* and population build-up bioassays, the total number of eggs laid, the number of viable eggs, the proportion of eggs that were non-viable, and total number of nymphs per plant were examined using univariate GLM. The development of nymphs in the population build-up bioassay was examined using a multivariate GLM with the proportions of 1st, 2nd, and 3rd instars as dependent variables. Data from the experiment with aposymbiotic and symbiotic planthoppers was analyzed using two-factor GLM. 

For all analyses, post hoc Tukey tests were applied to all significant factors with >2 levels. Residuals were plotted following all GLMs and, where these were not normal or homogenous, the data was transformed using log(data + 1) or arcsine (for proportions) transformations. Where data could not be homogenized, we ranked the data. Transformations including ranking of data are indicated together with the results. All analyses were conducted using SPSS version 23.0 (IBM SPSS Statistics).

## 3. Results

### 3.1. Adaptation to IR62 after 20+ Generations of Continuous Rearing

Planthoppers developed faster, had greater longevity, and laid more eggs on TN1 compared to IR62 (Table 1). They also produced more honeydew when feeding on TN1, and this was primarily derived from phloem feeding. When exposed to IR62 plants, planthoppers that had been continuously reared for 20+ generations on IR62 developed more rapidly, attained a greater body weight, produced more eggs, and had a greater amount of phloem-derived honeydew as a proportion of total honeydew compared to non-virulent planthoppers on the same resistant host (Table 1). IR62-selected nymphs also had higher densities of YLS compared to non-virulent planthoppers; however, this was mainly due to very high densities (mean = 6144 YLS mg^−1^) when feeding on the resistant host (Table 1).

Planthoppers that had been continuously reared on IR62 grew larger and developed more quickly irrespective of exposed host, but these also had lower nymph survival and laid fewer eggs (Table 1). Relatively low honeydew production and a high proportion of xylem-derived honeydew by both virulence-adapted and avirulent planthoppers suggests that the resistant host plant continued to reduce the feeding efficiency of the virulence-adapted planthoppers; however, the virulence-adapted planthoppers did produce more phloem-derived honeydew than the avirulent planthoppers when feeding on IR62 (Table 1).

### 3.2. Effects of Adapted Male and Female Parents on Progeny Fitness

**Figure 2:**
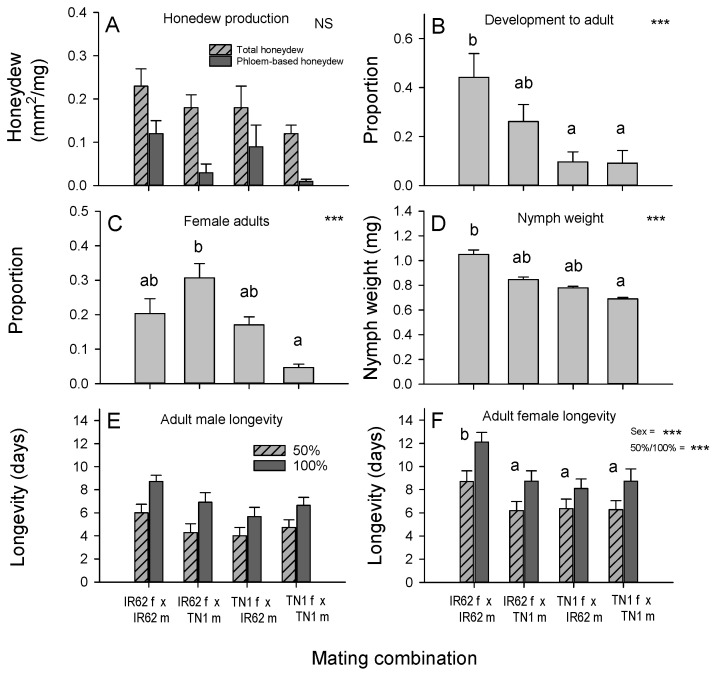
Performance of planthopper nymphs (**A**–**D**) and adults (**E**,**F**) derived (F1) from controlled mating of virulent and non-virulent parents when feeding on the resistant rice variety IR62. Performance was compared by recording (**A**) honeydew production, (**B**) nymph development, (**C**) proportion of adults that were female, (**D**) nymph weight, (**E**) the time to 50% and 100% mortality of adult males, and (**F**) the time to 50% and 100% mortality of adult females (i.e., longevity). Standard errors are indicated (N = 12). NS = no significant parent effect, *** = highly significant parent effect (*p* < 0.001). Lowercase letters indicate homogenous groups (Tukey *p* > 0.05). Data in (**A**) was ranked, (**B**,**C**) arcsine-transformed, and (**D**) log-transformed before analyses.

There was no statistically significant effect of parents on the amount of honeydew produced by nymphs (F_3,44_ = 1.964, *p* = 0.133) or the area of filter paper cover by phloem-derived honeydew as a proportion of the total area of honeydew produced when feeding on IR62 (F_3,44_ = 1.349, *p* = 0.271: Figure 2A).

The virulence of parents had no effect on nymph survival on IR62 (79 ± 3%) (survival: F_3,44_ = 1.562, *p* = 0.212). Development to adult was greater for F1 (IR62 ♀ × IR62 ♂) nymphs than F1 (TN1 ♀ × TN1 ♂) nymphs (proportion as adults: F_3,44_ = 5.667, *p* = 0.002: Figure 2B). More females emerged from populations with an IR62 parent than from the TN1 ♀ × TN1 ♂ matings (females: F_3,44_ = 10.534, *p* < 0.001: Figure 2C). The resulting planthopper biomass was also greater for F1 (IR62 ♀ × IR62 ♂) nymphs than F1 (TN1 ♀ × TN1 ♂) nymphs (planthopper biomass: F_3,44_ = 4.303, *p* < 0.010) (Figure 2D). 

Adult mortality increased over the course of the adult survival bioassay (time: F_1,88_ = 152.644, *p* < 0.001) (Figure 2E,F). Females lived for longer than males (sex: F_1,88_ = 261.475, *p* = 0.001) and adults of both sexes from the F1 (IR62 ♀ × IR62 ♂) progeny lived longer than progeny from the other mating combinations (F_3,88_ = 19.168, *p* < 0.001: Figure 2E,F). There was a significant time × parent interaction (F_3,88_ = 5.017, *p* = 0.045) because adults without an IR62-adapted parent survived fewer days between 50% and 100% mortality than adults from the other mating combinations (Figure 2E,F). All other interactions were non-significant.

**Figure 3:**
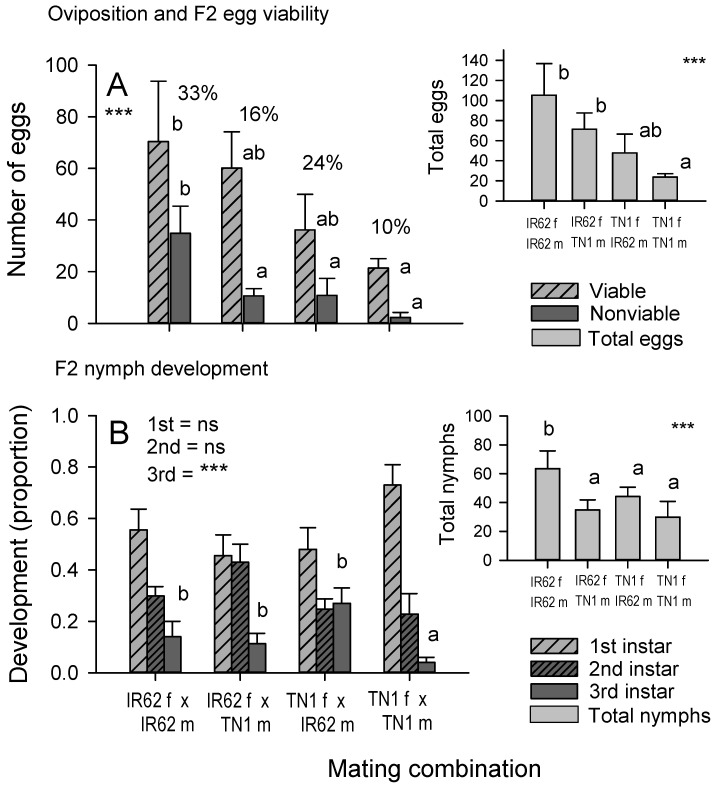
(**A**) Oviposition and (**B**) population development from adults (F1) produced through controlled mating of virulent and non-virulent parents when feeding on the resistant rice variety IR62. Inset graphs indicate the total number of eggs (**A**) and total number of nymphs (**B**) derived from mated pairs. Note that eggs and nymphs are F2 individuals. Standard errors are indicated (N = 12). NS = no significant parent effect, *** = highly significant parent effect (*p* ≤ 0.001). Lowercase letters indicate homogenous groups (Tukey *p* > 0.05). Egg and nymph numbers were log-transformed and proportions arcsine-transformed before analyses.

More eggs were laid by planthoppers with a virulent female parent than planthoppers with two avirulent parents (eggs: F_3,44_ = 5.723, *p* = 0.002: Figure 3A inset). However, a higher proportion of the F2 (IR62 ♀ × IR62 ♂) eggs were non-viable compared to eggs from all other mating combinations (proportion of eggs that were non-viable: F_3,44_ = 6.937, *p* = 0.001: Figure 3A). Overall, more viable eggs were laid by F1 (IR62 ♀ × IR62 ♂) females than by F1 (TN1 ♀ × TN1 ♂) females (viable eggs: F_3,44_ = 4.524, *p* = 0.008: Figure 3A).

More F2 nymphs were produced on IR62 plants by F1 (IR62 ♀ × IR62 ♂) adults than by F1 (TN1 ♀ × TN1 ♂) adults in the population build-up bioassay (number of nymphs: F_3,44_ = 4.950, *p* = 0.005: Figure 3B inset). Relative proportions of nymphal instars differed between mating treatments (instars: Wilk’s Lambda = 0.560, F_9,102_ = 3.056, *p* = 0.003). Proportions of 1st and 2nd instars were similar across treatments (1 instar: F_3,44_ = 1.603, *p* = 0.202; 2 instar: F_3,44_ = 1.695, *p* = 0.182), but there were fewer 3rd instars on plants with F1 (TN1 ♀ × TN1 ♂) adults (3 instar: F_3,44_ = 6.665, *p* = 0.001: Figure 3B).

### 3.3. YLS Densities and Planthopper Virulence

**Figure 4:**
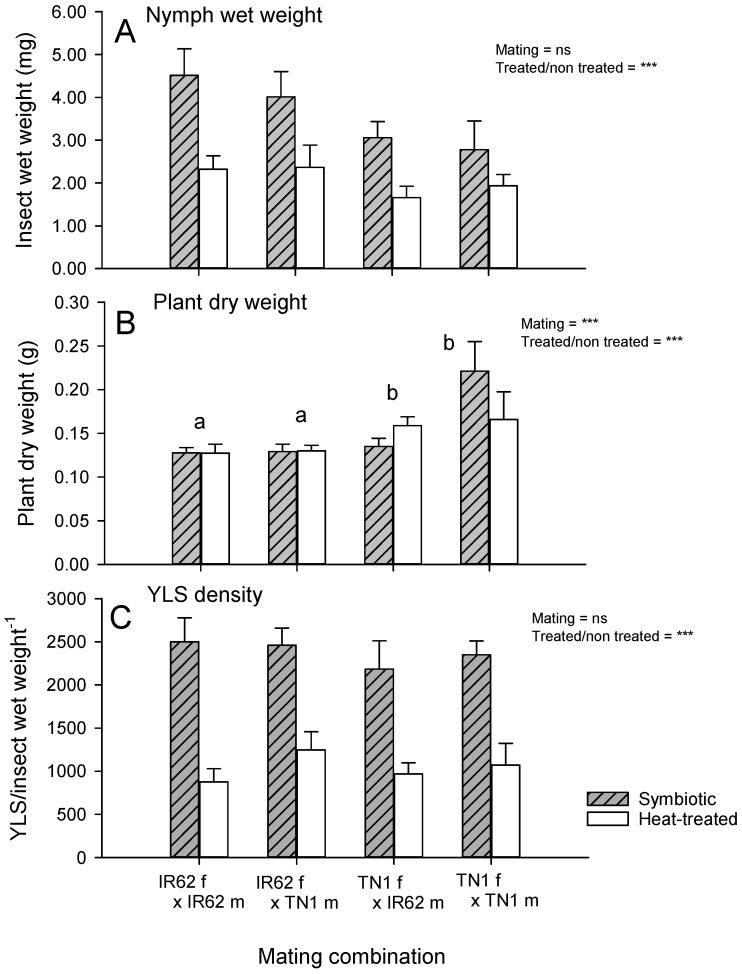
(**A**) Wet weight of unmated adult females (F1) derived from controlled mating between virulent and avirulent planthoppers with (**B**) the corresponding final weights of IR62 seedlings after planthopper feeding. (**C**) The estimated densities of YLS at the end of the experiment are also presented. Gray bars represent symbiotic planthoppers and white bars represent aposymbiotic planthoppers. Standard errors are indicated (N = 12). NS = no significant effect, *** = highly significant effect (*p* < 0.001). Lowercase letters indicate homogenous groups (Tukey *p* > 0.05). Data were log-transformed before analyses.

Planthopper weights were similar from all mating combinations (wet weight: F_3,72_ = 1.987, *p* = 0.124), but weights were lower for aposymbiotic (heat-treated) planthoppers (F_1,72_ = 19.619, *p* = 0.001: Figure 4A). At the end of the experiment, seedlings were larger after feeding by F1 (TN1 ♀ × TN1 ♂) and F1 (TN1 ♀ × IR62 ♂) planthoppers compared to seedlings attacked by F1 (IR62 ♀ × IR62 ♂) or F1 (IR62 ♀ × TN1 ♂) planthoppers (plant dry weight: F_3,72_ = 6.251, *p* = 0.001), irrespective of whether the planthoppers were heat-treated or not (interaction: F_1,72_ = 0.289, *p* = 0.592: Figure 4B). Mating combination had no effect on the density of YLS (YLS density: F_3,72_ = 0.960, *p* = 0.416). Heat treatment reduced YLS densities across mating treatments (F_1,72_ = 54.548, *p* = 0.001: Figure 4C). All interactions were non-significant.

## 4. Discussion

We predicted that if virulence is derived entirely from the female parent, then planthoppers with a virulent female parent would demonstrate a similar capacity to feed and develop on a resistant exposed host irrespective of whether the male parent was virulent or not (Figure 1A). We found evidence that virulence against IR62 (*BPH3* or *BPH32*-based resistance) is partly female-derived, but that some of the virulence is also derived from the virulent male parent. Nevertheless, the females contributed more than the males to virulence in the progeny. We also predicted that by reducing YLS densities through heat treatment, female-derived virulence would no longer be observed, thereby indicating that virulence factors are vertically transmitted through YLS in the egg (Figure 1D). However, we found only weak evidence to support a role for YLS density in virulence adaptation: virulent planthopper nymphs had higher densities of YLS when feeding on IR62 compared to virulent planthoppers on TN1 (Table 1). Furthermore, heat-treated planthoppers had similar growth irrespective of the virulence of the parents, while there was a tendency for control planthoppers with virulent female parents to grow larger on IR62 relative to planthoppers descended from avirulent females (Figure 4A)—thus, supporting our predictions. However, IR62 seedlings that were exposed to planthoppers were still smaller where planthoppers had a virulent female parent, regardless of whether the planthoppers were heat-treated or not (Figure 4B), indicating that YLS density is only weakly related to female-derived virulence. Our study, therefore, corroborates previous studies that have indicated a functional role for YLS in planthopper nutrition [23,24,27] and a likely greater functional contribution to feeding on resistant compared to susceptible rice [7,18], but we found no clear evidence for a direct relation between YLS density and virulence adaptation. 

### 4.1. The Nature of Virulence

Virulence adaptation in rice planthoppers and leafhoppers is a major limitation to the utility of resistant rice varieties across Asia [1,4,8,9]. Recent studies suggest that many identified resistance genes and gene loci are currently ineffective against target planthopper and leafhopper populations in many regions [33,36]. Among the major donor varieties for resistance, only a few remain widely resistant to planthoppers and these are mainly varieties with ≥2 resistance genes [33,36]. We used IR62 in our experiments after screening several resistance donors and modern resistant varieties. The variety IR62 was among the most resistant varieties, had largely stable resistance (expressed throughout growth and development), and appears to show consistent resistance based upon deployment records from the Philippines and on the continuing avirulence of Philippines planthopper populations [34]. Avirulent planthoppers feeding on IR62 have low survival, slow development, smaller adults, and lay fewer eggs than planthoppers on susceptible varieties (Table 1). However, when exposed to IR62 under laboratory conditions, individual planthoppers begin to adapt behaviorally to the host’s defenses (by, for example, improving feeding efficiency over time [37]). Ferrater and Horgan (2016) [38] found that conspecific nymphs feeding together on the same IR62 plant can induce susceptibility in the plant resulting in heavier nymphs of both virulent and avirulent planthoppers. Furthermore, Horgan et al. (2016) [37] found that ca 80% of the non-adapted nymphs that survive feeding on IR62 are females and, among these, between 20–50% can have swollen abdomens—indicating potentially high reproductive output [37]. Nevertheless, egg laying and population build-up are often severely inhibited on IR62, even where planthoppers appear otherwise adapted to feed and grow on the variety—as seen in this study (Table 1) and indicated in a further selection study using the same IR62-planthopper system [39]. These observations suggest that virulence adaptation results from a combination of adapted traits working on different aspects of the planthopper’s complete life cycle. For example, research by Kobayashi et al. (2014) [11,15] and Jing et al. (2014) [16] used similar study systems but different research methods to identify a single recessive gene controlling virulence as measured through honeydew production [15], or several genes for virulence related to other life-history traits based on association mapping [16].

We found that the continuous rearing of planthoppers on IR62 over 20+ generations improved planthopper fitness (survival × reproduction) on the resistant host, but that the adaptation of certain life-history traits was incomplete (i.e., partial adaptation); for example, adapted planthoppers continued to have slower development and laid fewer eggs on the resistant host than on the susceptible host (Table 1). Most notably, feeding in the selected planthoppers involved a considerably higher consumption of xylem-based fluids than is normally observed for planthoppers feeding on susceptible varieties (Table 1). Therefore, although the selected planthoppers had adapted to access and feed from the host’s phloem, feeding was fundamentally different because of high xylem consumption. A similar observation has been made for ‘adapted’ planthoppers feeding on rice with the *BPH1* gene [13]. Indeed, Ferrater et al. (2015) [14], who monitored honeydew composition in five planthopper populations during selection over 20 generations on IR62, PTB33, and IR65483 (containing the *BPH10* gene for resistance), found that xylem feeding remained consistently high throughout selection on these hosts, even though other fitness traits (including weight gain and oviposition) had improved over the course of selection. The apparent mismatch between honeydew production and the ultimate survival and development in planthoppers on resistant rice may be due to planthopper consumption of xylem fluids to dilute toxic defense compounds or to rehydrate during periods of low nutrient acquisition [28,40,41]. Furthermore, honeydew bioassays are normally conducted for 24 or 48 h, but planthoppers on resistant rice can improve their feeding efficiency over time and gradually reduce xylem feeding [37]. Associated with selection for virulence in IR62, we noted that the survival of nymphs from the selected populations was marginally lower than in avirulent populations and male longevity was also lower—indicating possible trade-offs with virulence. In a related study, Horgan et al. (2020) [39] found that IR62-selected planthoppers were capable of feeding on a range of varieties with other resistance genes and were less selective of rice varieties (whether resistant or susceptible) in general; furthermore, the selected planthoppers often had relatively high resistance to Fipronil but low resistance to Imidacloprid and BPMC. Such trade-offs may underlie the durability of IR62 and its resistance genes (*BPH3* and *BPH32*).

### 4.2. Yeast-like Symbionts in Virulence Adaptation

Compared to planthoppers (virulent and avirulent) feeding on TN1, we found that virulent planthopper nymphs reared on IR62 had more than double the densities of YLS in their bodies (Table 1). Furthermore, the greater survival of female nymphs on IR62 (Figure 2C) is consistent with higher densities of YLS in pre-adult females [22,26]. Comparative YLS densities were, however, very similar among adult planthoppers (avirulent and virulent) feeding on IR62 in our final experiment (Figure 4). In the study of selection by Ferrater et al. (2015) [14], YLS densities in adult female planthoppers were also similar between virulent and avirulent planthoppers on IR62 and TN1, respectively. Evidence suggests that YLS play a greater role in the nutrition of nymphs than adults [17,23,27], which might explain why we failed to observe the same trends in YLS densities in our experiments with nymphs and adults. Nevertheless, our results with nymphs are consistent with the idea that YLS play a greater role in host nutrition on the resistant host. Lu et al. (2004) [18] found that YLS abundance increased over time as planthoppers ‘adapted’ to the resistant varieties IR26 (*BPH1* gene) and ASD7 (*BPH2* gene). Their study monitored YLS during only four generations, and it is possible that densities would have continued to increase had populations been further selected. In another study, YLS densities in five PTB33-selected colonies increased over 20 generations of selection and were significantly higher than for avirulent planthoppers from five related colonies reared on TN1, despite monitoring densities using adult females [14]. The highest YLS densities were recorded when the PTB33-selected planthoppers were capable of high oviposition rates on the resistant variety. In the same study, YLS densities declined dramatically in populations that were continuously reared on the highly susceptible variety IR22 [14]. Therefore, our results corroborate previous research to suggest that YLS densities are affected by selection on resistant rice varieties, but the degree to which selection affects YLS densities and/or YLS functions varies considerably depending on the rice host.

Our experiment with aposymbiotic planthoppers weakly supported our predictions for the role of YLS density in virulence adaptation. Whereas aposymbiotic planthoppers performed similarly on IR62 irrespective of the virulence of their parents, symbiotic planthoppers with virulent female parents appeared better adapted to feed on the IR62 plants (Figure 4A). However, the trend was weak, which may be due to our using adults in the experiment. Although YLS densities are highest in adult females prior to oviposition, these YLS likely play a less important role in facilitating feeding and digestion at this life stage [25]. Based on the final weights of seedlings in the experiment, plant resistance still reduced seedling damage where the planthoppers had avirulent, aposymbiotic female parents (Figure 4B). Nevertheless, our results from this and other bioassays still suggest that females contribute more to virulence adaptation than males (see below), thereby preserving the possibility that YLS or other endosymbionts, including bacterial symbionts that are vertically transmitted through the egg [31,42], might provide specific functions (unrelated to YLS densities) that allow planthopper progeny to overcome the defenses of resistant rice. 

### 4.3. Role of Female versus Male Parent in Virulence Adaptation

Because YLS and other endosymbionts are mainly transmitted to progeny in the egg [22,25,29,30], we expected that if YLS determined virulence, then progeny with a virulent female parent would also be virulent against IR62. With only weak evidence for a role of YLS density in virulence through comparisons with aposymbiotic virulent planthoppers (Figure 4), our predicted trends can only identify female-mediated virulence—without further elucidation of probable mechanisms. Such female-mediated mechanisms might involve other endosymbionts, including bacterial and other fungal symbionts (that are more heat-tolerant than YLS), cytoplasmic factors, or sex-linked virulence genes. The results from our bioassays were generally consistent. Nymph development, the dry weight of nymphs, adult longevity, the number of eggs laid, and the population size of progeny on IR62 plants all followed the same trends (Figure 2 and Figure 3) with greatest fitness apparent in progeny derived from a virulent male and female, and successively lower fitness, firstly where only the female parent was virulent, and secondly where only the male parent was virulent. Finally, planthoppers without virulent parents had the lowest fitness on IR62. The same trends were apparent, but not statistically significant, for honeydew production by progeny (Figure 2A) and the wet weights of adult progeny (Figure 4A). These results indicate that virulence is at least partially associated with the female parent, but that the male also contributes to overall virulence.

Because we assessed virulence using parameter averages from progeny populations, we cannot assess whether virulence might be associated with dominant or recessive traits (i.e., mendelian genetics). However, the observed patterns do suggest that virulence is associated with factors derived from the male and/or female parent, together with some other factor(s) derived only from the female. This is consistent with evidence from genetic studies of resistance conducted by Kobayashi et al. (2014) [15] and Jing et al. (2014) [16] that together indicate multiple genetic mechanisms underlying planthopper virulence to *BPH1* resistance in rice. Our results are also consistent with several studies that suggest that endosymbionts, passed vertically through the egg, promote the adaptation of planthoppers to resistant rice or to toxic compounds in the planthopper’s environment [17,19,43,44]. Furthermore, the results of our experiments demonstrate that virulence adaptation can be partial because it involves several virulence mechanisms, it involves both the male and the female parent, and likely involves both the planthopper and its mutualist symbionts. Partial resistance is most pronounced during virulence adaptation in leafhoppers—for example, adaptations for leafhopper (*Nephotettix virescens* (Distant)) nymphs to feed and develop on resistant rice can take as little as 5 generations, but adaptations to lay eggs on resistant varieties can take a further 10–15 generations of selection [45]. Our planthoppers also demonstrated partial adaptation to IR62 (they survived and developed well but were inefficient feeders and had slow population growth). Partial virulence may be of little benefit to planthoppers and leafhoppers that cannot build up populations in the field because of poor egg-laying capacity on a resistant host—as demonstrated in this and other studies [39]. However, even partial resistance seems to be stable in populations that are returned for several generations to a susceptible host [9,46,47]. An understanding of how the different components of virulence are passed to progeny, and how these might be altered through crop production practices [43,48,49], could aid in the management of virulence on resistant rice.

### 4.4. Improving Research Designs

Our experiments were conducted under greenhouse conditions. Recent research has shown that planthoppers on IR62 at suboptimal temperatures appear unaffected by the host’s resistance. For better selection, planthopper colonies should be maintained—and experiments conducted—at optimal temperatures (about 25 °C [50]). We used large colonies to delay inbreeding and, because larger numbers of adapted individuals can be selected from large colonies to initiate subsequent generations, the average virulence of the colonies appears more stable over time compared to smaller colonies [10,14]. Therefore, we recommend that such large colonies also be used in future selection studies in favor of the smaller colonies used in some previous studies [14,28]. YLS and other symbionts constitute a diverse community with several ecological functions [19,20,31,42,45,51,52]. Future studies might better determine the relative species compositions of the symbiont communities in progeny and their functional capacity using molecular tools. Finally, several near-isogenic rice lines with clearly identified resistance genes are now available, and the resistance mechanisms associated with many of these genes have been largely determined [45,53,54]. Such near-isogenic lines can be used to select planthoppers and leafhoppers with the purpose of identifying virulence genes in planthoppers or their symbionts that target specific resistance genes in the rice host.

## 5. Conclusions

Virulence against IR62 in the brown planthopper involves a complex of traits that encompass a series of behavioral and physiological mechanisms. Virulence is derived from both the male and female parents; however, the female contributes more than the male to progeny virulence. This is consistent with the transovarial transmission of virulence factors to progeny, including specific functional genes in endosymbionts. We found only weak evidence that YLS densities in adults contribute to virulence, but strong evidence that YLS are essential for normal planthopper development on the resistant host and that relatively high YLS densities occur in virulent nymphs exposed to host resistance factors. Further research is required to improve the deployment of resistance genes in farmer’s fields such that complete virulence to durable genes such as *BPH3* and *BPH32* is avoided.

## Figures and Tables

**Table 1:** Baseline parameters for virulent (natal host = IR62) ^1^ and avirulent (natal host = TN1) ^1^ planthoppers following exposure to IR62 and TN1 hosts. Means ± standard errors are presented.

Exposed Host	TN1		IR62		F-Exposed Host ^5^	F-Natal Host ^4^	F-Interaction ^5^
Natal Host	TN1	IR62	TN1	IR62			
Proportion survival ^2^	0.88 ± 0.05	0.71 ± 0.08	0.88 ± 0.05	0.72 ± 0.09	0.06 ns	0.06 *	0.02 ns
Proportion adults ^2^	0.38 ± 0.09	0.73 ± 0.09	0.07 ± 0.05	0.53 ± 0.10	11.60 ***	14.93 ***	0.30 ns
Adult ♀ weight (mg) ^3^	0.95 ± 0.05	1.04 ± 0.08	0.43 ± 0.08	1.05 ± 0.10	9.60 ***	19.36 ***	10.28 ***
Adult ♂ longevity (days)	11.83 ± 0.39	10.18 ± 0.49	6.63 ± 0.71	8.73 ± 0.53	56.01 ***	0.60 ns	21.01 ^6,^***
Adult ♀ longevity (days)	15.83 ± 0.96	13.60 ± 0.47	8.73 ± 1.06	12.10 ± 0.84			
Number of eggs laid ^3,7^	368.00 ± 14.02	287.30 ± 14.80	36.80 ± 3.33	128.70 ± 23.50	17.62 ***	9.16 **	5.19 *
Phloem-derived honeydew (mm^2^/mg) ^4^	0.66 ± 0.13	0.55 ± 0.05	0.01 ± 0.01	0.12 ± 0.05	121.03 ***	1.95 ns	2.18 ns
Xylem-derived honeydew (mm^2^/mg) ^4^	0.01 ± 0.01	0.01 ± 0.01	0.11 ± 0.02	0.12 ± 0.04	54.46 ***	1.73 ns	0.30 ns
Total honeydew (mm^2^/mg) ^4^	0.77 ± 0.11	0.82 ± 0.08	0.09 ± 0.01	0.21 ± 0.04	72.57 ***	0.01 ns	1.41 ns
Phloem/total honeydew ^4^	0.97 ± 0.03	0.98 ± 0.05	0.07 ± 0.05	0.39 ± 0.13	81.62 ***	4.29 *	1.56 ns
YLS density (1000 s/mg)	3.34 ± 0.32	2.96 ± 0.47	3.08 ± 0.07	6.14 ± 1.11	4.07 *	5.05 *	7.20 **

^1^: Colonies (A and B) with IR62 as natal host and colony B with TN1 as natal host were at generations 20–25 at the time of testing. ‘Colony’ (i.e., A or B) was initially included in analyses as a nested factor but had no effect on any parameter and was later removed. ^2^: Arcsine-transformed before analysis. ^3^: Log(X + 1)-transformed before analysis. ^4^: Ranked before analysis. ^5^: DF = 1,44 (N = 12) for all parameters except ‘number of eggs’ where DF = 1,36 (N = 10), and adult longevity (male and female) where DF = 1,88 (N = 12); ns = *p* > 0.05, * = *p* ≤ 0.05, ** = *p* ≤ 0.01, *** = *p* ≤ 0.001. ^6^: Adult sex (F_1,88_ = 39.905 ***) and natal host × exposed host interaction (F_1,88_ = 21.005 ***) also affected longevity (other interactions were non-significant). ^7^: Egg laying was monitored for IR62 ♀ + IR62 ♂ and TN1 ♀ + TN1 ♂ adults.

## Data Availability

The data presented in this study are available on request from the corresponding author.
